# Low Serum retinol-binding protein-4 levels in acute exacerbations of chronic obstructive pulmonary disease at intensive care unit admission is a predictor of mortality in elderly patients

**DOI:** 10.1186/1476-9255-10-31

**Published:** 2013-10-07

**Authors:** Qihui Jin, Yueliang Chen, Yufeng Lou, Xiaojun He

**Affiliations:** 1 Department of Geriatric Medicine, The Second Affiliated Hospital of Zhejiang University School of Medicine, Hangzhou, Zhejiang Province, China; 2 Department of ICU, Sir Run Run Shaw Hospital of Zhejiang University School of Medicine, Hangzhou, Zhejiang Province, China; 3 Department of Clinical Laboratory, The First affiliated Hospital of Zhejiang University School of Medicine, Hangzhou, Zhejiang Province, China; 4 Department of Emergency Medicine, Editorial Board of the Chinese Journal of Emergency Medicine, The Second affiliated Hospital of Zhejiang University School of Medicine, Hangzhou, Zhejiang Province, China

**Keywords:** Acute exacerbations of chronic obstructive pulmonary disease, Retinol-binding protein-4, Predictor, Elderly, Intensive care unit

## Abstract

**Background:**

Acute exacerbations of chronic obstructive pulmonary disease (AECOPD) are thought to be associated with increased mortality in elderly patients. Low retinol-binding protein-4 (RBP4) is associated with a high risk of respiratory infections in the general population. Therefore, we hypothesized that low RBP4 levels are associated with an increased risk of AECOPD and can be used as a biomarker for AECOPD in elderly patients.

**Methods:**

Enzyme-linked immunosorbent assays were used to assess RBP4 levels in elderly with AECOPD within the first 24 hours after intensive care unit admission. Forty-six elderly patients with stable COPD in outpatient clinics and 50 healthy elderly persons who had physical examinations as outpatients were controls.

**Results:**

In AECOPD patients, RBP4 levels were lower than those in stable COPD patients and healthy controls (59.7 vs 91.2 and 113.6 mg/L, *p* < 0.001). RBP4 levels were decreased by 30.6% in non-survivors compared with survivors (51.5 vs 74.2 mg/L, *p* < 0.001). A higher Acute Physiology and Chronic Health Enquiry II (APACHE II) score and Simplified Acute Physiology score (SAPS II) were associated with lower RBP4 levels (r = −0.692, *p* = 0.024 and r = −0.670, *p* = 0.015, respectively). RBP4 was positively correlated with creatinine and body mass index, and negatively correlated with C-reactive protein and Global Initiative for Chronic Obstructive Lung Disease stage. Multivariate logistic regression showed that RBP4 was an independent mortality predictor of AECOPD (odds ratio: 0.926, *p* = 0.007). Analysis of the area under the receiver operating characteristic (AUC) curve showed that RBP4 showed good discrimination (AUC: 0.88; 95% confidence interval: 0.78–0.94; *p* = 0.008) in predicting mortality. RBP4 improved the prognostic accuracy of mortality for the APACHE II and SAPS II scores.

**Conclusions:**

Serum RBP4 levels are significantly reduced in elderly AECOPD patients. RBP4 might be a good predictive biomarker for mortality in elderly AECOPD patients in the intensive care unit.

## Background

Chronic obstructive pulmonary disease (COPD) is a condition characterized by progressive airflow limitation, which causes considerable morbidity and mortality worldwide, and results in substantial social and economic burden [1]. Patients with acute exacerbations of chronic obstructive pulmonary disease (AECOPD), particularly elderly people, have high mortality rates and usually need admission to intensive care units (ICUs) [2]. Mortality rates of aged patients with AECOPD in ICU can be up to 50% [3]. The risk of 90-day mortality is three times as great in elderly patients admitted with AECOPD as in younger patients [4]. Many factors have been identified as predictors of ICU mortality in patients with COPD [5], but the predictive value of the clinical parameters varies in different studies.

Recently, considerable attention has been paid to adipocytokines, biologically active factors secreted by adipose tissue as an endocrine organ [6]. Several hormones secreted by adipose tissue have been identified to be involved in acute critical illness [7]. Adipose tissue is an important inflammatory source because of cytokines produced from the adipocyte itself, as well as owing to infiltration by proinflammatory macrophages [8]. Adipose tissue is a potent producer of inflammatory mediators and may contribute to systemic inflammation in COPD [9]. Adipokines are associated with the systemic inflammatory process during exacerbations of COPD [10].

Retinol-binding protein-4 (RBP4) is a molecule found in the circulation, thought to be secreted mainly by adipose tissue and the liver, and is a specific transporter for retinol in the circulation [11]. RBP4 has been identified as an adipokine involved in detection of insulin resistance and type 2 diabetes [12]. Recently, RBP4 has been extensively studied and is associated with various pathologies [13,14]. However, no studies have investigated the relationship between RBP4 and AECOPD.

This study aimed to identify whether RBP4 is predictive for mortality among various risk factors in elderly AECOPD patients in the ICU. We report here that lower levels of plasma RBP4 have a higher risk of mortality and can be used for predicting prognosis in elderly AECOPD patients in the ICU.

## Methods

### Patients

COPD was defined as dyspnea, chronic cough, or sputum production and/or a history of exposure to risk factors for the disease, and post-bronchodilator 1-second forced expiratory volume/forced vital capacity (FEV_1_/FVC) < 70% according to the Global Initiative for Chronic Obstructive Lung Disease (GOLD) guidelines [15]. Pulmonary function testing was performed in a hospital pulmonary function laboratory by trained technicians according to American Thoracic Society and European Respiratory Society standards and guidelines [16]. Mild, moderate, and severe COPD were defined based on GOLD guidelines as follows. Mild COPD was defined as an FEV_1_/FVC ratio < 0.7 and FEV_1_ % ≥80% predicted. Moderate COPD was defined as an FEV_1_/FVC ratio < 0.7 and FEV_1_ % 50–79% predicted. Severe COPD was defined as an FEV_1_/FVC ratio < 0.7 and FEV_1_ % 30–49% predicted. Very severe COPD was defined as an FEV_1_/FVC ratio < 0.7 and FEV_1_ % < 30% predicted.

Patients with AECOPD admitted to the ICU must meet at least one of the following indications [17]: (1) severe dyspnea that responds inadequately to initial emergency therapy; (2) changes in mental status (confusion, lethargy, coma); (3) persistent or worsening hypoxemia (PaO_2_ < 5.3 kPa, 40 mmHg), and/or severe/worsening of hypercapnia (PaCO_2_ > 8.0 kPa, 60 mmHg), and/or severe/worsening of respiratory acidosis (pH < 7.25) despite supplemental oxygen and noninvasive ventilation; (4) the need for invasive mechanical ventilation; (5) hemodynamic instability and the requirement for vasopressors. Exclusion criteria included interstitial pneumonia, asthma, pulmonary tuberculosis, bronchiectasis, pulmonary fibrosis, and lung cancer according to clinical history and data [18]. Arterial blood gas analysis was completed immediately at ICU admission.

Systemic inflammatory response syndrome (SIRS) according to international, standardized criteria [19] was characterized by the presence of at least two of the following four clinical criteria: (1) fever or hypothermia (temperature > 38.0°C or < 36.0°C); (2) tachycardia (> 90 beats/min); (3) tachypnoea > 20 breaths/min or < 32 mmHg, or the need for mechanical ventilation support; (4) altered white blood cell (WBC) count (> 12 000 cells/μl or < 4000 cells/μl) or the presence of > 10% band forms. Sepsis was defined as SIRS with an infection based on the 1991 American College of Chest Physicians/Society of Critical Care Medicine Sepsis Directory [20].

According to the above criteria, a total of 100 patients admitted to the First and Sir Run Run Shaw Hospitals from September 2010 to February 2012 were recruited. Clinical data within the first 24 hours after ICU admission were collected. All patients were aged ≥65 years, with an ICU stay of ≥72 hours. All patients were scheduled for a follow-up visit until being discharged from the ICU (survivors) or they died in the ICU (non-survivors). To further analyze the prognostic role of RBP4 for AECOPD, we subdivided the patients according to whether they had type 2 diabetes mellitus (T2DM) or sepsis. T2DM was diagnosed according to American Diabetes Association criteria [21], and sepsis was defined during first 24 hours at the ICU admission.

Forty-six stable (no exacerbations for at least 4 weeks prior to study entry) [18] COPD patients who had normal blood counts, liver enzymes, and C-reactive protein (CRP) levels in outpatient clinics were included and 50 non-COPD healthy elderly people determined by physical examination as outpatients served as controls.

Written informed consent was obtained from each participant or his or her spouse, and the study was approved by the local ethics committee (ethics committee of the First and Sir Run Run Shaw Hospitals, Zhejiang University, Zhejiang, China).

### Sample and data collection

Routine blood analyses, including WBC, total bilirubin (TB), γ-glutamyl transferase, albumin (ALB), blood urea nitrogen, serum creatinine (sCr), lactic acid (LA), CRP, B-type natriuretic peptide (BNP), procalcitonin (PCT), RBP4, and blood and sputum culture were immediately performed after admission to the ICU. RBP4 serum concentrations were measured by enzyme-linked immunosorbent assay according to the manufacturer’s instructions (Shanghai Resun Biotechnology Limited Company, Shanghai, China). BNP was measured by the electrochemiluminescence technique (cobas h 232 system, Roche, Germany). PCT was measured by enzyme link fluorescence assay (mini-VIDAS, automatic immune fluorescence analyzer, France). The homeostasis model assessment for insulin resistance (HOMA-IR) was used as a measurement of insulin resistance and was calculated as fasting plasma glucose (mmol/L) × fasting serum insulin (mU/mL)/22.5. The Acute Physiology and Chronic Health Evaluation (APACHE) II score, and Simplified Acute Physiology Score (SAPS II) were evaluated within the first 24 hours (day 1) after ICU admission.

### Data analysis

For statistical analysis, measurements of RBP4, ALB, LA, PCT, and CRP were logarithmically transformed to obtain normal distributions (Kolmogorov-Smirnov test, *p* > 0.05). Data are presented as mean and standard deviation, or median and interquartile range, as appropriate. Fisher's exact was used for comparison of the primary disease between the non-survivor group and the survivor group. For comparison of study variables between the two groups, the Student’s *t*-test or Mann–Whitney *U* test was performed. Pearson’s correlation coefficients were calculated to evaluate the relationship between serum RBP4 levels and other clinical and metabolic variables. We assessed the risk factors for ICU mortality using univariate analysis and multiple logistic regression analysis to determine independent factors and dependent factors for prognosis.

We compared the number of risk factors for the death probability of AECOPD. Discrimination was calculated using area under the curve (AUC) values. The AUC values were compared using a nonparametric approach. AUC analysis was also used to calculate the cut-off values, sensitivity, specificity, and overall correctness. Finally, cut-off points were calculated by calculating the best Youden index (sensitivity+specificity-1). Cumulative survival curves as a function of time were plotted using the Kaplan–Meier approach and were compared using the log rank test. A *p* value < 0.05 was considered statistically significant. All statistical analyses were performed using SPSS 15.0 (SPSS, Chicago, IL).

## Results

### RBP4 serum levels are significantly reduced in AECOPD patients compared with stable COPD patients and healthy controls

The median serum concentration of RBP4 in stable COPD was 91.2 mg/L and in healthy controls it was 113.6 mg/L. AECOPD patients had significantly reduced serum RBP4 concentrations compared with stable COPD patients and healthy controls (median, 59.7 vs. 91.2 and 113.6 mg/L, *p* < 0.001; Table 1). AECOPD patients had a lower body mass index (BMI) compared with stable COPD patients and healthy controls (median, 20.7 vs. 21.6 and 23.4, *p* < 0.01). Serum RBP4 levels were different between male and female patients.

**Table 1:** Comparison among ICU AECOPD patients, stable COPD patients, and healthy controls

**Parameter**	**AECOPD**	**Stable COPD**	**Healthy controls**	***p *value**
Number	100	46	50	
Sex (male/female)	72/28	34/12	34/16	ns
Age median (range)(yr)	76 (66–91)	75 (65–89)	75 (65–86)	ns
BMI median (range)(m^2^/kg)	20.7 (17.2-26.4)	21.6* (17.8-27.5)	23.4** (19.5-26.8)	< 0.01
RBP4 median(range)(mg/L)	59.7 (21.3-128.6)	91.2* (42.1-343.9)	113.6** (57.2-369.2)	< 0.001

BMI, body mass index; RBP4, retinol-binding protein-4. ns, not significant; *p < 0.01, AECOPD vs stable COPD; **p < 0.001, stable COPD vs healthy controls.

### Descriptive characteristics of the patients

A total of 100 AECOPD patients meeting ICU admission standards [22] were included in this study. The median age was 76 years and 26% were females. In 54 patients (54%), SIRS with evidence of underlying infection was found. The principal diagnoses in these patients included diabetes (n = 26), coronary heart disease (n = 57), hypertension (n = 58), cerebrovascular disease (n = 43), renal failure (n = 21), and respiratory failure (n = 76). The median length of stay in the medical ICU was 12 days. Risk assessment on admission to the ICU showed a median APACHE II score of 24 points and a median SAPS II score of 40. Spirometry confirmed that all patients had moderate to very severe COPD (GOLD II-IV). Mean FEV_1_ was 40% predicted (range, 24–66%), and the mean FEV_1_/FVC ratio was 45% (28–63%).

A total of 64 patients (48 males, 16 females) who died were included in the non-survivor group and 36 patients (26 males, 10 females) who survived were included in the survivor group. The distributions of sex, age, primary diseases (diabetes, coronary heart disease, hypertension, cerebrovascular disease, and renal failure), ICU days, pulmonary function, and blood gas analysis tests were not significantly different between the two groups. Premorbid diseases have not been shown to be predictive of outcome. The durations of AECOPD, APACHE II and SAPS II scores, mechanical ventilation, and ventilation time were significantly elevated in the non-survivor group compared with the survivor group (*p* < 0.05 or < 0.01, Table 2).

**Table 2:** Baseline characteristics of 100 patients presenting with AECOPD to the ICU within the first 24 hours

**Parameter**	**All patients**	**Survivors**	**Non-survivors**	***p *value**
Number	100	36	64	
Sex(male/female)	72/28	26/10	48/16	0.761
Age median (yr)	76 (66–91)	74 (66–88)	77 (66–91)	0.799
Duration of COPD (mo)	201 (18–350)	199 (18–328)	204 (22–350)	0.611
Duration of AECOPD (d)	8 (1–17)	6 (1–16)	9* (2–17)	0.004
ICU days median (d)	12 (3–28)	11 (3–28)	13 (5–28)	0.073
FEV_1_ (%predicted)	40 (24–66)	43 (28–66)	38 (24–64)	0.224
FVC (% predicted)	71 (47–85)	74 (49–85)	68 (47–81)	0.218
FEV_1_/FVC ratio (%)	45 (28–63)	48 (30–63)	43 (28–60)	0.418
PH	7.37 (7.21-7.52)	7.40 (7.21-7.49)	7.36 (7.21-7.52)	0.781
PaO_2_ (mm Hg)	63 (54–88)	66 (57–88)	62 (54–84)	0.556
PaCO_2_ (mm Hg)	65 (38–88)	68 (42–84)	63 (38–88)	0.218
Severity of COPD				
GOLD stage II (moderate), n (%)	14(14%)	12(33.33%)	2(3.13%)	< 0.001
GOLD stage III (severe), n (%)	60(60%)	20(55.56%)	40(62.5%)	< 0.001
GOLD stage IV (very severe), n (%)	26(26%)	4(11.11%)	22(34.37%)	< 0.001
APACHE-II score	24 (18–38)	22 (18–37)	25** (19–38)	0.001
SAPS II score	40 (22–62)	38 (22–60)	42** (24–70)	0.001
BMI (m^2^/kg)	20.7 (17.2-26.4)	20.9 (18.3-26.0)	20.6 (17.2-26.4)	0.072
Caloric intake (kcal/d)	1274 (922–1689)	1418 (1220–1689)	1100** (922–1425)	0.001
SIRS, n (%)	71(71%)	20(55.56%)	48(75%)*	0.045
Sepsis, n (%)	54(54%)	12(33.33%)	42(65.63%)**	0.002
White blood cells (10^9^/L)	12.3 (1.8-32.2)	12.5 (2.0-30.6)	12.2 (1.8-32.2)	0.073
BNP (pg/ml)	546 (176–1208)	455 (176–1010)	557 (221–1208)	0.069
Total bilirubin (μmol/L)	22.5 (4.2-55.7)	21.7	23.5* (5.4-55.7)	0.047
		(4.2-43.4)		
Serum albumin (g/L)	3.1 (2.2-4.4)	3.2 (2.6–4.4)	3.0 (2.2–3.8)	0.173
Serum creatinine (mg/dl)	1.3 (0.5–3.2)	1.2 (0.6–3.1)	1.3 (0.5–3.2)	0.279
Lactic acid (mmol/L)	2.2 (0.8-5.3)	2.1 (0.8-4.7)	2.3* (0.8-5.3)	0.038
C-reactive protein (mg/dl)	49 (15–229)	52 (34–203)	48 (15–229)	0.066
Procalcitonin (μg/L)	2.8 (0.5-15.7)	2.7 (0.5-12.4)	2.9 (1.7-15.7)	0.051
RBP-4 (mg/L)	59.7 (21.3-128.6)	74.2 (32.6-128.6)	51.5** (21.3-84.2)	0.001

ICU, intensive care unit; FEV_1_, 1-second forced expiratory volume; FVC, forced vital capacity; APACHE, acute physiology and chronic health evaluation; SAPS, simplified acute physiology score; BMI, body mass index; SIRS, systemic inflammatory response syndrome; BNP, B-type natriuretic peptide; RBP4, Retinol-binding protein-4. Compared with Survivors, *p < 0.05, **p < 0.01.

### Comparison between survivors and non-survivors

**Figure 1:**
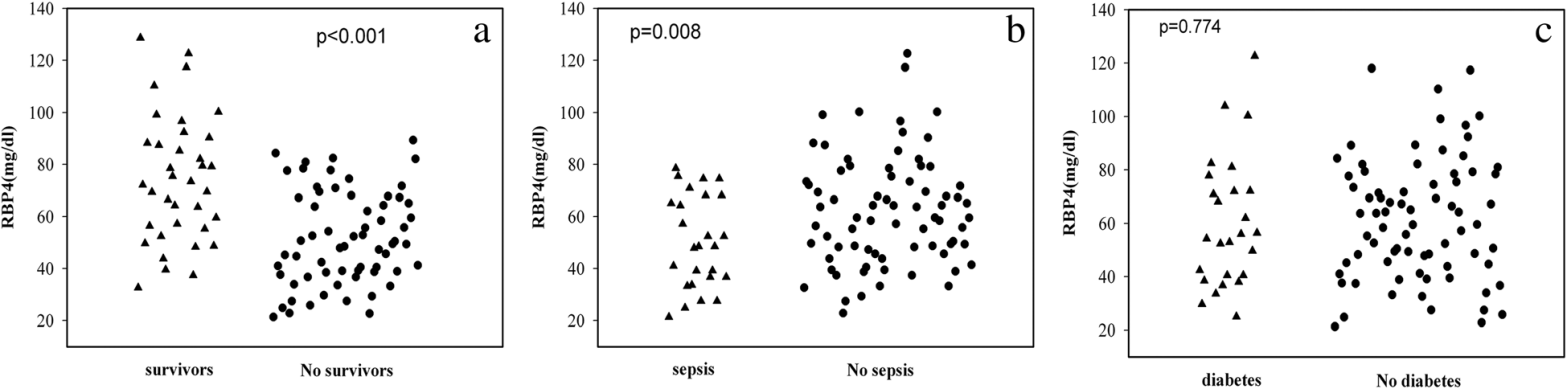
**RBP4 levels in AECOPD patients among the different groups. (a)** RBP4 levels in survivors and non-survivors. **(b)** RBP4 in patients with sepsis and those without sepsis. **(c)** RBP4 levels in patients with diabetes and those without diabetes.

We investigated whether serum RBP4 concentrations upon ICU admission can predict clinical outcome and mortality. Interestingly, patients who died during ICU hospitalization (64/100) displayed significantly lower serum RBP4 concentrations than patients who survived (mg/L; 51.5 [21.3–84.2] vs 74.2 [32.6–128.6], *p* < 0.001) (Figure 1a). In survivors, differences were more pronounced in the APACHE II score (*p* = 0.008), the SAPS II score (*p* = 0.005), LA (*p* = 0.032), TB (*p* = 0.044), duration of AECOPD (*p* = 0.037), and ventilation time (*p* = 0.001) compared with non-survivors. By contrast, WBC (*p* = 0.06), CRP (*p* = 0.08), PCT levels (*p* = 0.24), and FEV_1_ (*p* = 0.224) were similar in the survivor and non-survivor groups. In addition, BMI (*p* = 0.072), metabolic parameters on admission, including glucose (*p* = 0.57), and ALB levels (*p* = 0.33) were not different between survivors and non-survivors (Table 2). Severe COPD (GOLD III–IV) mortality rate was 72.1%. The higher the GOLD grade, the higher the mortality rate, with the risk of mortality related to the GOLD classification [23]. RBP4 levels were negatively correlated with GOLD stage by Pearson’s correlation (r = −0.34, *p* = 0.001).

### RBP4 levels in AECOPD patients with sepsis and those without sepsis

**Figure 2:**
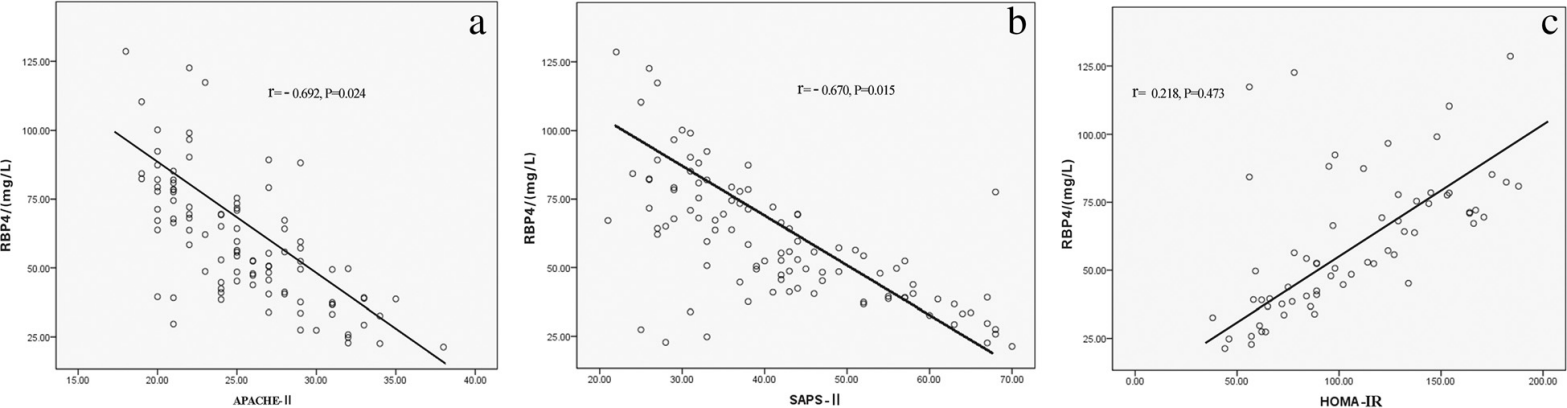
**Correlations between RBP4 levels and the APACHE II score, the SAPS II score, and HOMA-IR. (a)** Correlation between RBP4 levels and the APACHE II score (r = −0.692, *p* = 0.024). **(b)** Correlation between RBP4 levels and the SAPS II score (r = −0.670, *p* = 0.015). **(c)** Correlation between RBP4 levels and HOMA-IR (r = 0.218, *p* = 0.473).

AECOPD is frequently associated with SIRS, which evolves into septic shock and has a high mortality. To investigate the role of RBP4 levels in AECOPD with sepsis, patients were classified into those with sepsis and those without sepsis. We found that AECOPD patients with sepsis had significantly lower RBP4 levels compared with patients without sepsis (mg/L; 47.4 [21.9–82.4] vs 83.1 [23.3–128.6], *p* = 0.008) (Figure 1b). In all patients, RBP4 levels decreased with increased severity of the disease. High-risk patients in the highest quartile of the APACHE II score and in the highest quartile of the SAPS II score had significantly lower RBP4 concentrations (r = −0.692, *p* = 0.024 and r = −0.670, *p* = 0.015) (Figure 2a, b).

### RBP4 levels in AECOPD patients with diabetes and those without diabetes

In our study, DM was not a crucial factor of prognosis in AECOPD patients (Table 2). RBP4 levels were not different between patients with and those without preexisting T2DM on admission to the ICU (mg/L; 72.2 [24.7–128.6] vs 68.6 [21.3–119.2], *p* = 0.774) (Figure 1c). RBP4 concentrations in patients with diabetes were not correlated with insulin sensitivity as calculated by the HOMA-IR (r = 0.473, *p* = 0.218, Figure 2c).

### Factors affecting RBP4 levels

**Figure 3:**
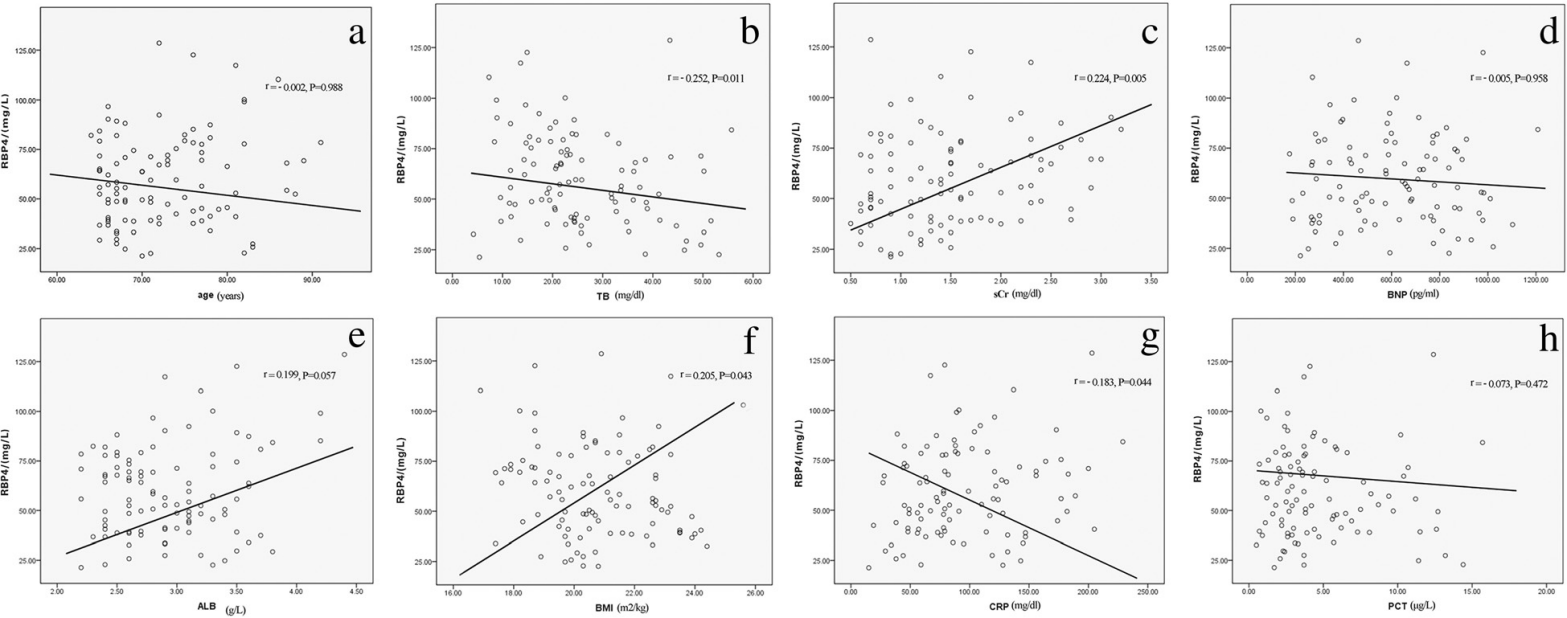
**Correlations between RBP4 levels and other risk factors. (a)** Correlation between RBP4 levels and age (r = −0.002, *p* = 0.988). **(b)** Correlation between RBP4 levels and TB (total bilirubin) (r = −0.252, *p* = 0.011). **(c)** Correlation between RBP4 levels and sCr (r = 0.224, *p* = 0.005). **(d)** Correlation between RBP4 levels and BNP (r = −0.005, *p* = 0.958). **(e)** Correlation between RBP4 levels and ALB (r = 0.199, *p* = 0.057). **(f)** Correlation between RBP4 levels and BMI (r = 0.205, *p* = 0.043). **(g)** Correlation between RBP4 levels and CRP (r = −0.183, *p* = 0.044). **(h)** Correlation between RBP4 levels and PCT (r = −0.073, *p* = 0.472).

RBP4 levels were not correlated with age in AECOPD (r = −0.002, *p* = 0.988) (Figure 3a). RBP4 levels were associated with liver, renal, and heart function. Liver function was identified as a strong predictor of RBP4 because RBP4 levels were directly correlated with parameters indicating the liver’s biosynthetic capacity, such as TB (r = −0.252, *p* = 0.011) (Figure 3b). RBP4 levels were also correlated with markers of renal failure, specifically sCr (r = 0.224, *p* = 0.005) (Figure 3c), but they were not correlated with BNP (r = −0.005, *p* = 0.958) (Figure 3d) and ALB (r = 0.199, *p* = 0.057) levels (Figure 3e). BMI had a significant effect on RBP4 levels in AECOPD patients (r = 0.205, *p* = 0.043) (Figure 3f). There was an inverse correlation between RBP4 levels and CRP levels (r = −0.183, *p* = 0.044) (Figure 3g), while no correlation between RBP4 and PCT levels was found (r = −0.073, *p* = 0.472) (Figure 3h).

### RBP4 is a prognostic marker of mortality in AECOPD patients

To assess the prediction of RBP4 on the prognosis of mortality of AECOPD patients, we performed univariate and multivariate logistic regression, which compared the prognostic values of RBP4 and other well known risk factors, including age, BMI, the APACHE II score, the SAPS II score, glucose levels, TB levels, WBC, LA, CRP levels, and PCT levels. As shown in Table 3, RBP4, LA, TB, the APACHE II score, and the SAPS II score were significant predictors of ICU mortality. Comparison among discriminatory values of the nine risk factors is also shown in Table 4. The discriminatory ability of RBP4 with the AUC was 0.88 (95% confidence interval [CI] [0.78–0.94)], *p* = 0.008), the APACHE II score with the AUC was 0.75 (95% CI [0.66–0.84], *p* = 0.036), and the SAPS II score with the AUC was 0.77 (95% CI [0.67–0.86], *p* = 0.028). AUC analysis showed that RBP4 has the best discriminatory power. A combined model including RBP4 and the APACHE II score (AUC: 0.75 vs 0.78, *p* = 0.042) improved the prognostic accuracy compared with the APACHE II score alone. Similarly, RBP4 tended to improve the prognostic accuracy of the SAPS II score in a combined model (AUC: 0.77 vs 0.81, *p* = 0.033).

**Table 3:** Prediction of mortality of AECOPD by univariate and multivariate logistic regression analyses

**Predictor**	**Univariate**			**Multivariate**		
	**OR**	**95% CI**	***p *value**	**OR**	**95% CI**	***p *value**
Sex	1.260	0.846–1.505	0.718	1.017	0.822–1.627	0.973
Age	1.099	0.979–1.235	0.110	1.028	0.836–1.412	0.209
BMI	1.355	0.776–1.634	0.247	1.177	0.735–1.709	0.466
APACHE II	2.112	1.872–3.991	**0.009**	1.978	1.607–4.212	**0.023**
SAPSII score	2.371	1.846–4.957	**0.004**	2.043	1.582–5.213	**0.012**
Glucose levels	1.367	0.942–1.832	0.251	1.209	0.841–2.034	0.418
Albumin	0.957	0.791–1.248	0.076	0.855	0.722–1.377	0.107
White blood cells	1.977	0.953–2.110	0.954	1.843	0.921–2.232	1.021
Total bilirubin	1.173	1.021–4.336	**0.032**	1.076	1.009–4.455	**0.048**
BNP	1.721	0.996–3.018	0.056	1.237	0.811–3.247	0.081
Lactic acid	1.315	1.223–7.753	**0.022**	1.202	1.176–7.802	**0.041**
CRP	1.413	0.993–3.034	0.189	1.244	0.928–3.149	0.224
Procalcitonin	1.759	0.964–3.021	0.051	1.611	0.872–3.178	0.084
RBP4	0.992	0.895–0.995	**0.001**	0.926	0.867–0.997	**0.007**

BMI, body mass index; APACHE, acute physiology and chronic health evaluation; SAPS, simplified acute physiology score; BNP, B-type natriuretic peptide; CRP, C-reactive protein; RBP4, retinol binding protein-4; CI, confidence interval.

**Table 4:** Receiver operating curves for the prediction of mortality (n = 64) in all 100 AECOPD patients on admission

**Parameter**	**AUC (95% CI)**	***p *value**
RBP4	0.88(0.78-0.94)	0.008
White blood cells	0.59(0.54-0.72)	0.069
C-reactive protein	0.62(0.58-0.77)	0.372
Total bilirubin	0.69(0.61-0.79)	0.057
BNP	0.66(0.52-0.81)	0.083
Lactic acid	0.73(0.51-0.83)	0.054
Procalcitonin	0.71(0.52-0.82)	0.096
APACHE II	0.75(0.66-0.84)	0.036
Combined model (APACHE II/RBP4)	0.78(0.71-0.88)	0.042
SAPS II	0.77(0.67-0.86)	0.028
Combined model (SAPS II/ RBP4)	0.81(0.71-0.91)	0.033

BNP, B-type natriuretic peptide; APACHE, acute physiology and chronic health evaluation; SAPS, simplified acute physiology score; AUC, area under the ROC curve; CI, confidence interval.

## Discussion

In this study, we showed that elderly AECOPD patients in the ICU had severe disease and high mortality, and RBP4 levels were lower in non-survivors than survivors. Lower RBP4 levels were accompanied by a higher mortality. For the subgroups, RBP4 serum levels were lower in patients with sepsis than in those without sepsis. RBP4 was an independent predictor of mortality of AECOPD in elderly patients in the ICU.

RBP4, the circulating transporter for vitamin A, is synthesized in the liver and adipose tissue. RBP4 levels in patients with AECOPD were significantly lower than those in stable COPD and healthy subjects, as anticipated from previous studies using the same assay [24]. RBP4 is elevated in T2DM and is linked to insulin resistance [12,14,22]. However, in our study, RBP4 levels were not different between patients with and those without preexisting T2DM on admission to the ICU. This may have been affected by therapy (for example, insulin, glucose and catecholamine infusions). In addition, AECOPD patients reach an advanced state of inflammation where RBP4 is negligible in insulin resistance [25].

Serum RBP4 levels were associated with nutritional status in COPD patients. Nutritional status is related to respiratory impairment and systemic inflammation in patients with AECOPD [19]. Malnutrition increases the incidence of complications and mortality, and can be identified as a risk factor associated with short-term mortality of elderly patients admitted for AECOPD [20]. Low BMI has been shown to be an independent predictor of in-hospital mortality [24]. In AECOPD patients, low BMI is associated with a higher degree of bronchial obstruction and pulmonary hyperinflation [17]. AECOPD patients have a high risk of malnutrition because of limited caloric intake and increased energy expenditure, and the use of mechanical ventilation altered catabolism [17]. Our results showed that BMI in AECOPD patients was significantly lower than that in stable COPD patients. Serum RBP4 levels were significantly lower in AECOPD patients with a worse nutritional status, and non-survivors’ daily intake of calories (1100 kcal/d) was significantly lower than the survivors’ intake (1418 kcal/d). We found that RBP4 levels were positively associated with BMI. Severe calorie restriction with weight loss reduces circulating RBP4 levels, and calorie restriction reduces adipose tissue messenger ribonucleic acid expression of RBP4 [23]. Reduction of RBP4 could be a protective mechanism to prevent the organism from developing insulin resistance in an acute inflammatory state and weight loss [26]. RBP4 expression increases with increasing adipose tissue mass and dietary intake of nutrients can modulate RBP4 concentrations [27]. There is a causal relation between serum RBP4 levels and adipose tissue mass [28,29]. RBP4 mRNA and protein expressions are high in overweight/obese people [30]. In different conditions, RBP4 genetic variants induce RBP4 expression changes in mRNA and protein levels [31]. Regulatory single-nucleotide polymorphism in the RBP4 gene modifies its expression in adipocytes, and RBP4 levels are correlated with insulin-mediated glucose uptake and are associated with BMI [32].

Multivariate logistic regression analysis showed that RBP4 was an independent predictor of mortality. In the assessment and management of critically ill patients, knowledge of prognostic factors is crucial to estimate the risk for mortality. Receiver operating curves for the prediction of mortality indicated that RBP4 with an AUC of 0.88 was higher than those of the clinical scores (APACHE II and SAPS II scores, AUC: 0.75 and 0.77). Our study shows that measurement of RBP4 levels has a high prognostic use as an early and independent risk predictor for potential death to complement and thereby improve the APACHE II and SAPS II scores. Aged patients with low serum RBP4 levels caused by AECOPD might have a poor prognosis.

The possible role of low RBP4 concentrations observed in the circulation of AECOPD patients is unclear. We consider that at least five reasons may explain this finding. The first reason might involve inflammation. RBP4 levels are reduced with individual stress levels of patients, and thus mirror the stress associated with the severity and extent of illness [8]. Tumor necrosis factor-α strongly down-regulates the production of RBP4 in primary human adipocytes [33]. Inhibition of gene expression and production of RBP4 by macrophages has been documented after exposure to bacterial endotoxins, such as *Escherichia coli* lipopolysaccharide [34]. We found that CRP and RBP4 levels were negatively correlated. A second reason might be insufficient daily calorie intake. Weight loss and severe calorie restriction promotes reduction in adipose tissue and plasma levels of RBP4 [23]. Third, the liver is the main source of RBP4 in humans, and the non-survivor group had significantly increased TB serum concentrations compared with the survivor group. Consequently, reduced serum RBP4 levels in patients are closely associated with liver function [35]. Fourth, critically ill or aged patients are more likely to have vitamin A deficiency. Patients with inflammatory diseases might benefit from vitamin A supplementation during inflammatory injury. Retinoic acid plays a protective role against tumor necrosis factor-α-induced lung injury [36]. Fifth, theoretically, an increase in RBP4 consumption, or removal increased by extravasation due to capillary leakage or increased metabolic clearance [14], may play a role.

## Conclusions

Serum RBP4 levels in AECOPD patients are correlated with liver and kidney functions, nutritional status, and disease stage, but not serum HOMA-IR, and are an independent factor of overall survival in elderly AECOPD patients. In the ICU, AECOPD patients with RBP4 deficiency have a greater risk for short-term hospital mortality, and require more intensive surveillance. In addition, we should pay more attention to the function of RBP4 as a potential determinant of prognosis.

## Competing interests

The authors declare that they have no competing interests.

## Authors’ contributions

All authors helped to draft the manuscript and critically revise it. All authors agreed about the content of the paper and have read the manuscript and approved its submission. JQH and CYL conceived and designed the experiments. CYL and LYF collected the data. HXJ and JQH participated in statistical analysis.
